# Editorial: Education not cancelled: Pathways from absence to post-secondary education

**DOI:** 10.3389/fpsyg.2026.1870579

**Published:** 2026-06-26

**Authors:** Carolyn Gentle-Genitty, Christopher A. Kearney, David Heyne, Catriona O'Toole

**Affiliations:** 1 Butler University, Indianapolis, IN, United States; 2 University of Nevada, Las Vegas, NV, United States; 3 Deakin University, Burwood, VIC, Australia; 4 Maynooth University, County Kildare, Ireland

**Keywords:** school absence, compassion and collaboration in attendance, K-16 education, post-secondary academic performance, school attendance problems, unlearning attendance, youth and adult education

## A declaration, not a concession

The title of this Research Topic is itself a position statement. Education not canceled is not a reassurance, nor a platitude. It is a declaration: that however many days a student has missed, whatever roads have led them away from classrooms, and across whatever distances—geographic, emotional, economic—the door to learning has not been permanently closed. This Research Topic of international scholarship, published jointly in *Frontiers in Psychology* and *Frontiers in Education*, is offered in that spirit: not as an autopsy of failure, but as a map of possibility.

School absence—in all its forms—has long been framed as a problem of individual deficit: the anxious student, the disengaged family, the truant who chose not to show up. This framing has not served us well. What the research assembled here demonstrates, collectively and compellingly, is that absence is most often a signal—a symptom of misalignment between what schools offer and what students need. The educational path from absence to post-secondary engagement is neither straight nor easily traveled, but it exists. And it is our collective obligation to clear it.

## The scale of the challenge

Chronic absence has reached historically elevated levels in the post-pandemic period, with more than 6.5 million students in the United States alone missing 10% or more of school days. In Indiana and the broader Midwest, regional K-12 administrators consistently identify attendance as the number one challenge their schools face. The problem is not uniquely American: contributors to this volume document parallel crises from Chile, Germany, Korea, the Netherlands, Belgium, and Ireland, underscoring that the structural conditions that push students out of school—poverty, instability, cultural disconnection, and punitive policy—are not local idiosyncrasies but global patterns demanding international responses.

Crucially, absence does not merely delay education—it threatens to foreclose it entirely. The research of Contreras-Villalobos et al., examining dropout in youth and adult education in Chile, illustrates this with uncomfortable clarity: students who drop out of mainstream schooling and re-enter youth and adult education programs face a secondary dropout rate of 24%—a compounding effect that strips educational continuity from those already most vulnerable. School abandonment, the authors stress, is not simply a measure of student motivation; it is a product of institutional resource scarcity, inadequate teacher-to-student ratios, and organizational cultures that do not adequately sustain human connection.

## Naming what we see: the importance of classification

A recurring theme across this Research Topic, and across the broader scholarship of the editors, is the urgent need for precision in how we conceptualize and categorize school attendance problems. The field has suffered from conflation: truancy, school refusal, withdrawal, and exclusion are not interchangeable terms, yet they are frequently collapsed into undifferentiated “absenteeism,” with interventions applied in similarly undifferentiated ways—and ineffectively.

The four-part classification system described my members of this editorial team—distinguishing truancy (unauthorized, often socially-motivated absence), withdrawal (family-centered, often logistical), exclusion (school culture-related, rooted in school policy and contributing to the student's experience of school as an unwelcoming place), and refusal (anxiety-driven, often with clinical features) is not taxonomic pedantry. It is the foundation for matched, effective intervention. A student whose anxiety keeps them home requires a fundamentally different response than a student whose family has prioritized seasonal work obligations. Treating both as “truants” and deploying the same enforcement-based response is, at best, ineffective; at worst, it deepens harm.

The contributions in this volume also illuminate the systemic conditions that produce exclusion-type absence: school environments characterized by discrimination, a lack of culturally responsive practice, and climates that signal to students from minoritized backgrounds that this place was not built for them. Enderle et al.s' qualitative case study of secondary students in Germany—exploring student-identified supports for overcoming attendance barriers—reinforces this finding from the student's own vantage point. Young people know what would help. We have not always thought about asking.

## Extending the horizon: the K-16 movement

One of the most significant conceptual contributions of this Research Topic is the work by Kearney et al. articulating a K-16 educational movement: a framework that identifies common mechanisms of disengagement and re-engagement across both K-12 and higher education systems. For too long, the literature on school attendance has treated the transition into post-secondary education as an endpoint—the finish line beyond which absence becomes someone else's concern. This is a costly fiction.

The structural forces that disengage students from high school do not cease at graduation. A student who struggled with exclusion-type absence in ninth grade does not automatically acquire the belonging, confidence, and navigational skills needed to thrive in a university lecture hall. The K-16 lens asks us to think developmentally and longitudinally—to trace pathways rather than count seat-time—and to build systems of support that travel with students across institutional transitions. This is the animating vision of the Research Topic as a whole.

## Attendance as belonging: a reorientation

Perhaps the most important reorientation this Research Topic demands is in the very question we bring to school attendance. The dominant institutional question—*Why are students absent?*—is a question about deviance from an expected norm. It locates the problem in the student. We propose a different question: *What conditions would make students want to stay?* This shift—from a compliance frame to a belonging frame—is not merely rhetorical. It transforms what schools look for, what they measure, and what they build.

The Gentle-Genitty Perception of School Social Bonding (PSSB) instrument, validated with over 3,500 students across diverse demographic groups, gives practitioners a concrete tool for operationalizing this question (Gentle-Genitty et al., 2025). By measuring four dimensions of school social bonding—attachment (trust in school adults), commitment (investment in school goals), involvement (participation in school life), and belief (alignment with school-related values)—the PSSB enables schools to locate precisely where belonging has been disrupted, and to design targeted responses. This moves attendance work from reactive policing to proactive relationship-building.

The contribution by O'Toole and Cirić on compassion, collaboration, and cultural responsiveness extends this belonging framework to its policy implications. Students who face attendance barriers do not move through a single intervention and emerge transformed. They require sustained ecosystems of support that account for their cultural identities, their family circumstances, and the institutional structures that may have already communicated, implicitly or explicitly, that they do not belong. True defense of educational access means auditing and dismantling the narrow, rigid messages about what education is for, as well as messages that signal to certain students and communities that they do not belong.

## Re-engagement, second chances, and the architecture of return

The work of Van Den Berghe et al. on “drop-in” students—those who return to education through second-chance programs—offers a nuanced portrait of re-engagement that resists easy optimism. Not all returns are completions. Profiles of drop-in students vary significantly: some are re-engaging with genuine momentum; others are cycling through systems that, despite good intentions, have not yet found the architectural features that sustain long-term participation. The finding that completion rates differ substantially by student profile has direct implications for how second-chance programs should be differentiated in their support structures.

The international scope of this Research Topic—spanning North America, South America, Europe, East Asia, and Australia—is not incidental. It reflects the editors' conviction that attendance problems, though locally instantiated, are globally patterned, and that solutions developed in one context carry transferable insights. The experience of Korean adolescents studying abroad and navigating re-entry, explored by Lee and Lee, speaks to the costs of educational discontinuity even in seemingly voluntary transitions—and invites us to consider what scaffolding students need to maintain educational identity across disruption.

## Implications for practice, policy, and research

The research assembled here is not merely descriptive. It is, at its core, an argument for transformation—of practice, policy, research, and institutional culture. For practitioners, the message is direct: interventions must be differentiated by type of attendance problem, informed by student voice, and grounded in relationship rather than enforcement. The 10-step attendance assessment process, the PSSB instrument, and the tailored intervention frameworks described across this volume give practitioners concrete pathways for doing this work responsibly.

For policymakers, the evidence demands attention to the structural determinants of absence: housing instability, poverty, discriminatory school climates, inadequate resources in youth and adult education, and punitive attendance policies that criminalize families rather than support them. The work of (Heyne et al. 2024) on recalibrating and, where necessary, radically overhauling attendance frameworks situates these policy questions in their proper scope. Incremental adjustment of existing systems will not suffice where those systems are themselves producing the exclusion we seek to address.

For researchers, this Research Topic identifies several frontiers worthy of sustained investigation: the mechanisms by which school bonding predicts post-secondary persistence; the comparative effectiveness of differentiated attendance interventions across cultural contexts; the long-term educational trajectories of students who enter through second-chance programs; and the design features of K-16 systems that most reliably sustain belonging across institutional transitions. The international network of scholars represented here—working through the International Network for School Attendance (INSA) and allied organizations—represents exactly the kind of collaborative infrastructure these questions require.

## A word about what this work asks of us

To say that education is not canceled is also to accept an obligation. If the door remains open, someone must hold it. That means school social workers who see the student behind the absence data. It means administrators who audit policies for the biases embedded within them. It means researchers who keep asking harder questions. It means policymakers who fund the second-chance structures that evidence shows can work—and who resist the temptation to measure educational success only by the first path taken.

The students whose absence this volume is ultimately about are not abstractions. They are students who have learned, often through direct experience, that institutions can fail them. What we are collectively proposing is that institutions can also be redesigned—to recognize, to include, to sustain. This Research Topic is offered as evidence that such redesign is possible, and as an invitation to the broader field to join in making it real.
